# A Critical Meta-Analysis of Lens Model Studies in Human Judgment and Decision-Making

**DOI:** 10.1371/journal.pone.0083528

**Published:** 2013-12-31

**Authors:** Esther Kaufmann, Ulf-Dietrich Reips, Werner W. Wittmann

**Affiliations:** 1 Institute of Education, University of Zurich, Zurich, Switzerland; 2 Department of Economics, Chair of Business and Economics Education II, University of Konstanz, Konstanz, Germany; 3 Department of Psychology, University of Konstanz, Konstanz, Germany; 4 Otto-Selz Institute for Applied Psychology, University of Mannheim, Germany; The Ohio State University, Center for Cognitive and Brain Sciences, Center for Cognitive and Behavioral Brain Imaging, United States of America

## Abstract

Achieving accurate judgment (‘judgmental achievement’) is of utmost importance in daily life across multiple domains. The lens model and the lens model equation provide useful frameworks for modeling components of judgmental achievement and for creating tools to help decision makers (e.g., physicians, teachers) reach better judgments (e.g., a correct diagnosis, an accurate estimation of intelligence). Previous meta-analyses of judgment and decision-making studies have attempted to evaluate overall judgmental achievement and have provided the basis for evaluating the success of bootstrapping (i.e., replacing judges by linear models that guide decision making). However, previous meta-analyses have failed to appropriately correct for a number of study design artifacts (e.g., measurement error, dichotomization), which may have potentially biased estimations (e.g., of the variability between studies) and led to erroneous interpretations (e.g., with regards to moderator variables). In the current study we therefore conduct the first psychometric meta-analysis of judgmental achievement studies that corrects for a number of study design artifacts. We identified 31 lens model studies (N = 1,151, k = 49) that met our inclusion criteria. We evaluated overall judgmental achievement as well as whether judgmental achievement depended on decision domain (e.g., medicine, education) and/or the level of expertise (expert vs. novice). We also evaluated whether using corrected estimates affected conclusions with regards to the success of bootstrapping with psychometrically-corrected models. Further, we introduce a new psychometric trim-and-fill method to estimate the effect sizes of potentially missing studies correct psychometric meta-analyses for effects of publication bias. Comparison of the results of the psychometric meta-analysis with the results of a traditional meta-analysis (which only corrected for sampling error) indicated that artifact correction leads to a) an increase in values of the lens model components, b) reduced heterogeneity between studies, and c) increases the success of bootstrapping. We argue that psychometric meta-analysis is useful for accurately evaluating human judgment and show the success of bootstrapping.

## Introduction

Improving judgment and decision making is of utmost importance across multiple domains of life, as even minor inaccuracies can sometimes have a major impact. For example, within the medical domain, if a physician is able to accurately diagnosis cancer, the patient will likely receive early treatment and has a greater chance to survive. Within other domains such as business or education, individuals (e.g., managers, teachers) must make important decisions over the use of human and financial resources based on their judgment of ambiguous situations (e.g., the payoff of a certain strategy, the intelligence of a student). Hence, it is no wonder that judgmental achievement and decision-making has for many years been an important area of research as reflected in the considerable number of studies which have evaluated the success of human judgment across multiple fields (e.g., [1]–[3]). Within judgment and decision-making approaches, the lens model ([4], see below) provides a useful framework for understanding and modeling components of judgmental achievement. Previous meta-analyses of lens model studies have indicated that estimates of judgmental achievement vary widely across studies (see [5]). Because previous meta-analyses [5], [6] have not corrected for methodological artifacts (e.g., measurement error), previous estimates of judgmental achievement are likely biased. Furthermore, there is ambiguity with regards to the extent to which heterogeneity in estimates of judgmental achievement across studies stems from methodological artifacts as opposed to ‘substantial’ differences due to underlying moderators (e.g., decision domain, judge expertise).

To address the problems with previous meta-analyses, we conduct a psychometric meta-analysis of lens model studies across a number of decision-making domains (e.g., business, medicine, education, psychology). We correct for multiple study design artifacts (e.g., sampling error, measurement error, dichotomization). We compare results of a traditional meta-analytical approach with the psychometric approach to examine how methodological artifacts bias estimates and may lead to erroneous interpretations. Furthermore, we examine the extent to which judgmental achievement varies by domain (e.g., if physicians judge more accurately than teachers), level of expertise (i.e., if experts judge more accurately than novices), and whether the effect of expertise differs by domain (i.e., if expertise leads to better accuracy in some domains but not in others).

Finally, a further goal of the current paper is to contribute to the development of better decision making tools. Researchers have used the lens model equation to build linear models to ‘bootstrap’ judges (that is, replace human judges by equations to guide decision making) to increase judgment accuracy. For example, researchers have built models that physicians can use to make important medical judgments (see for example [7]). Previous meta-analyses have suggested that bootstrapping judges generally results in a slight increase in judgmental achievement relative to human judgment, although there seems to be high heterogeneity in its success (e.g., [2], [6]). However, it is possible that failure to correct for methodological artifacts may have led to an over- or underestimation of the potential success of bootstrapping relative to human judges. We therefore examine whether psychometrically-corrected linear models for decision making can increase the success of bootstrapping.

### The Lens Model Framework

**Figure 1:**
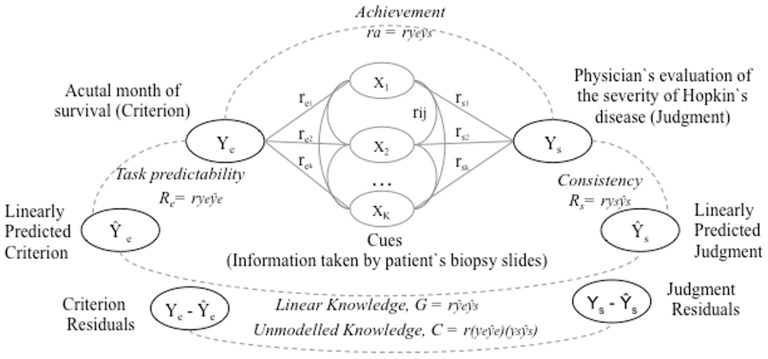
The lens model applied to physicians' diagnosis of cancer (see [8]).

The lens model [4] identifies multiple components of judgment (in) accuracy. In a typical lens model study, a ‘judge’ must make a number of decisions based on different pieces of information (‘cues’). Judgmental achievement is measured by the extent to which the judge's judgment matches (i.e., correlates) with an indicator of the actual outcome or situation (‘criterion’). Einhorn (second study, [8]) provides an example of a typical lens model study (see Figure 1). In this study, physicians evaluated the severity of Hodgkin's disease (cancer) based on patient's biopsy slides (see the right side of Figure 1, Y_s_). Physicians made a judgment with regards to the estimated survival time, which was compared with the actual number of months of survival (see the left side of Figure 1, Y_e_). A high correlation between physicians' judgments and the actual months of survival indicated high judgmental achievement.

The lens model is the basis for the lens model equation (*LME*; see [9]–[11]; for more background information on the *LME*, see [12]). As shown in Equation 1, the *LME* mathematically describes judgmental achievement (*r_a_*, i.e., the correlation between a person's judgments and a particular criterion) in terms of four components. Namely, judgmental achievement is equal to a *linear knowledge* term (*G*) multiplied by *task predictability* term (*R_e_*) term multiplied by a *consistency* term (*R_s_*) plus a *non-linear knowledge* term *(C)*. The linear knowledge component (*G*) refers to the correlation between the predicted human judgment and the predicted criterion (e.g., the predicted physician's judgment about survival time, the predicted actual months of survival). Task predictability (*R_e_*) refers to the multiple correlation of the cues with the criterion (e.g., the extent to which characteristics of the biopsy slide correlate with the months of survival), or in other words, the extent to which a decision can be made based on the information available. Consistency (*R_s_*) refers to the reliability of judgments, that is, the extent to which a judge reliably reaches the same decision based on the same pieces of information (e.g., the extent to which a physician reaches the same diagnosis based on biopsy slides with the similar characteristics), or in other words, the multiple correlation of the cues with the person's estimates. The non-linear knowledge component (*C*) represents the correlation between the variance not captured by the environmental predictability component or the consistency component (i.e., the correlation between the residuals from the above predictions). Previous research has revealed that the non-linear knowledge component is generally quite small (average *C* = .08, [13], p. 129); hence we exclude it from our analysis.

The definitions of the single components in detail are:

*r_a_* = the achievement index (i.e., the correlation between a person's judgments and the criterion),

*R_e_* = the task predictability index (i.e., the multiple correlation of the cues with the criterion),

*R_s_* = consistency (i.e., the multiple correlation of the cues with a judge's estimate),

*G* = a knowledge index that reflects achievement (i.e., the correlation between the predicted levels of the criterion and the predicted judgments), and

*C* = an unmodeled knowledge component that signifies the correlation between the variance not captured by the environmental predictability component or the consistency component (i.e., the correlation between the residuals from the above predictions).
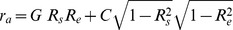
(1)


### The success of bootstrapping judges with a linear model

The lens model can be used to create linear judgment models (i.e., equations) that can be used to support judgment and decision making, essentially by ‘correcting’ for the inconsistency with which human judges use cues to reach a judgment. The process (and success) of replacing a human judge with a judgment model is referred to as ‘bootstrapping’ (see [6], [14]) and is also discussed under the topic of ‘man versus model of man’ (see [8]). The idea of creating such judgment models can be traced back to Meehl's [15] evaluation of whether clinical psychologists reach more accurate judgments about a patient relative to an equation.

Linear judgment models are defined with the same linear knowledge (*G*) and task predictability (*R_e_*) terms as in the lens model (see Equation 1), but with the assumption that there is perfect consistency in how a judge uses a particular piece of information (*R_s_* = 1), which is of course never the case with a human judge. As displayed in Equation 2, the success of a linear judgment model relative to a human judge can be estimated by the difference between the linear judgment model on the one hand and human judgmental achievement *r_a_* on the other hand (for details, see [2], p. 413):
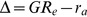
(2)


### Previous Meta-Analyses of Judgmental Achievement

Previous meta-analyses of lens model studies have revealed a large heterogeneity of judgmental achievement estimates across studies [5], [6] and that the success of bootstrapping judges with a linear judgment model generally results in only a slight increase in judgmental achievement (e.g., [2], [6]). However, to the best of our knowledge, no previous meta-analysis has followed a psychometric approach that appropriately corrects for multiple methodological artifacts. When left uncorrected, methodological differences between the studies included in the meta-analyses such as varying sample sizes (sampling error), varying reliability of the measurements used in different studies (measurement error), and dichotomization of a continuous variable can lead to biased estimations. Two previous meta-analyses of lens model studies (e.g., [5], [6]) applied ‘bare-bones meta-analysis’ (i.e., only correct for sampling error; [16], p. 132), but they did not control for other methodological artifacts. In the current study, we build on the results of previous bare-bones meta-analyses and follow the psychometric Hunter-Schmidt approach (see below) to correct for *multiple* study design artifacts and thus, we argue, arrive at less biased estimates of the *LME* components. We also check the robustness of our results by estimating the potential effect of publication bias, that is, the phenomenon for studies with significant results to be published more often relative to studies with non-significant results. In our case, it could be that studies with zero correlations are probably reported less frequently than studies with at least moderate correlations. Publication bias may thus threaten the representativeness of the studies included in the meta-analysis. We describe a new method for estimating potential publication bias (see *below*).

In the current study, we also extend previous research and investigate whether judgmental achievement varies according to judge expertise and decision domain. Karelaia and Hogarth [6] found that expertise is *negatively* related to judgmental achievement; however the authors did not control for decision domain. The authors concluded that expertise in some domains may be particularly difficult to develop and hence only weakly related to judgmental achievement (see also [17], [18]). Kaufmann and Athanasou [5] considered different decision domains, but they neglected to simultaneously consider judges' expertise. In the current psychometric meta-analysis, we therefore simultaneously investigate *both* expertise and decision domain as well as expertise within domains as potential moderators of judgmental achievement. Does expertise matter more in some domains relative to others? Finally, we also compare the success of bootstrapping (see Equation 2) with linear judgment models based on estimates of the *LME* components generated from bare-bones meta-analysis with the success of bootstrapping with linear judgment models based on estimates generated from psychometric meta-analysis.

## Methods

### Description of the Database

**Figure 2:**
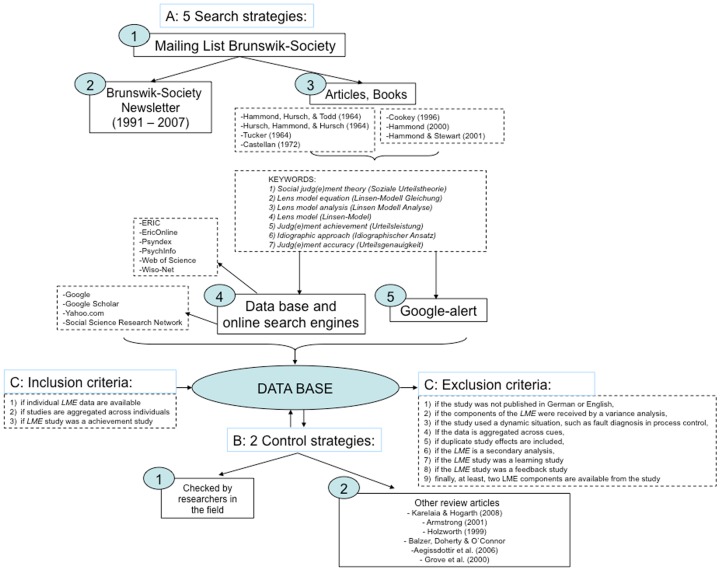
The process of identifying relevant studies for the meta-analysis.

The flowchart in Figure 2 depicts the five literature search strategies used in the current study (see Figure 2, point A). To find studies, we searched relevant databases (e.g. PsycINFO, Psyndex, Web of Science) using different keywords (e.g., ‘lens model’, ‘lens model equation’, ‘judgmental achievement’) as well as key articles and books in the area of research and activated a Google alert to notify us of any new relevant publications. We then cross-checked the database with sources found in other reviews (e.g., [19], see point B in the flowchart).

Point C lists the exclusion criteria. To prevent any aggregation bias, we only considered studies on judgment that had aggregated results across individuals, thus excluding those with aggregated results across cues (e.g., [20]). We included data derived from lens model studies of individual judges and of aggregated data across judges. We observe that the idiographic approach is often neglected in lens model studies [21]. Hence, mostly aggregated judgments made by multiple judges as opposed to judgments of single judges are reported in lens model studies.

In the current study we were interested in evaluating judgmental achievement *without any feedback opportunities* as would be the case in naturalistic, everyday settings. Business managers, for example, receive little feedback on the accuracy of their judgments. Moreover, they often can have no idea whether the feedback they do in fact receive is accurate or not (see [22]). Likewise, physicians frequently do not get any feedback about the accuracy of their judgments, as patients fail to return or are referred elsewhere, or diagnoses remain uncertain [23]. We therefore excluded studies in which judges received ongoing feedback on the accuracy of their decisions and/or had the opportunity to learn during the tasks. We argue that studies that included feedback and/or learning opportunities do not adequately represent the daily life of participants and could thus have biased our results.

Further details on the construction of our database, such as our search protocol, are available in Kaufmann [13].

A total of 31 studies met our inclusion criteria [8], [14], [23]–[51]. The studies were coded based on certain characteristics (e.g., year of publication, sample size) or possible moderator variables (judges' level of expertise, decision domain). Tables 1 and 2 summarize the characteristics of the included studies. Decision domain was coded as medicine, business, psychology, education, or as miscellaneous. With the exception of the medical domain, all other domains included both experts and non-experts (i.e., students) as judges. The database included 49 judgment tasks with 1,151 judgments made by 1,055 participants. Of the 1,055 participants, 68 participated in more than one task. Compared to the database by Kaufmann and Athanasou [5] our database is slightly different due to improved analysis tools and additional studies (e.g., [51]).

**Table 1:** Study characteristics ordered according to decision domain and expertise.

Study		Number of judges	Number of judgments	Number of cues	Judgment task	Criterion	Results
*a)*	*Medical science, expert:*						
1)	Nystedt, Magnusson [23]	4 clinical psychologists	38 patient protocols	3	Evaluate patients on three traits: *I:* Judgment on intelligence, *II:* Judgment on ability to establish contact,	Rating on three psychologist tests	*I: r_0_* = .63, *II: r_0_* = .66, *III: r_0_* = .47, (*, +)
					*I:* Judgment on intelligence, *III:* Judgment on control of affect and impulses	psychologist tests	
2)	Levi [24]	9 nuclear medicine physicians	280 patient cases, 60 replications	5	Assess probability of significant coronary artery disease	Coronary angiography	*r_s_* = .47 (*)
3)	LaDuca, Engel, Chovan [25]	13 physicians	30 patient profiles	5	Degree of severity (Congestive heart failure)	A single physician's judgment (▴)	*r_0_* = .62 (*)
4)	Smith, Gilhooly, Walker [26]	40 general practitioners	20 case profiles	8	Prescription of an antidepressant	Guideline expert (▴)	*r_0_* = .53
5a)	Einhorn^1^, *Second study* [8]	3 pathologists	*III:* 193 biopsy slides	9	Evaluate the severity of Hodgkin's disease	Actual number of months of survival	*III: r_0_* = −.001
5b)	Einhorn, *First study* [8]	29 clinicians	*I:* 77 MMPI profiles, *II:* 181 MMPI profiles	11	Judging of the degree of neuroticism- psychoticism	Actual diagnosis	*I: r_0_* = .16, *II: r_0_* = .19 (*, +)
6)	Speroff, Connors, Dawson [27]	123 physicians: 105 house staff, 15 fellows, 3 attending physicians	440 intensive care unit patients	32	Patients' hemodynamic status (Physicians' estimation)	The patient's actual hemodynamic status	*r_c_* = .42
*b)*	*Business science, experts:*						
7)	Ashton [28]	13 executives, managers, sales personnel	42 cases in a booklet	5	Predictions of advertising sales for *Time* magazine	Actual advertising pages sold	*r_s_* = .75 (*, +)
8)	Roos, Doherty [29]	16 agency managers	200/160 profiles	64/5	Predictability of success of life insurance salesman	One-year criterion for success	*r_0_ = *.13 (*, +)
9)	Goldberg [14]	43 bank loan officers	60 large industrial corporations profiles	5	Bankruptcy experience	Actual bankruptcy experience	*r = *.51
10)	Kim, Chung, Paradice [30]	3 experienced loan officers	119 financial profiles:	7	To judge whether a firm would be able to	Actual financial data	*I: r_0_* = .53
			*I:* 60 big firms,		repay the loan requested		*II: r_0_* = .58
			*II:* 59 small business firms				(*, +)
11)	Mear, Firth [31]	38 professional security analysts	30 financial profiles	10	Predicted security returns	Actual security returns	*r = *.12
	*Students:*						
12)	Wright [32]	47 students	50 securities profiles	4	Price changes for stocks (from 1970 until 1971)	Actual financial data	*r* = .22 (*, +)
13)	Harvey, Harries, (Experiment 1) [33]	24 psychology students	40 profiles	Not known	Forecast sales outcomes	Actual sales outcome	*r_0_ = *.98
14)	Singh [34]	52 business students	35 profiles	Not known	Estimates of the stock of a company	Actual realized values	*r_0_ = *.84
*c)*	*Educational science, experts:*						
15)	Cooksey, Freebody, Davidson [35]	20 teachers	118 profiles of kindergarten children	5	*I:* Reading comprehension *II:* Word knowledge	*I-II:* Actual end-of-year scores of each student on the two tests (▴)	*I: r_c_* = .56, *II: r_c_* = .57 (*, +)
	*Students:*						
16)	Wiggins, Kohen [36]	98 psychology graduate students	110 profiles	10	Forecast first-year-graduate grade point averages	Actual first-year-graduate grade point averages	*r_0_ = *.33
17)	Athanasou, Cooksey [37]	18 technical and further education students	120 student profiles	20	Deciding that students are interested in learning	Actual level of students' interest	*r_0_ = *.31 (*, +)
*d)*	*Psychological science, experts:*						
18)	Szucko, Kleinmuntz [38]	6 experienced polygraph interpreters	30 polygraph protocols	3-4	Truthful/untruthful responses	Actual theft	*r_pb_* = .23 (*, +)
19)	Cooper, Werner [39]	18: 9 psychologists, 9 case managers	33 inmates' data forms	17	Forecast violence during the first 6 months of incarceration.	Actual violent behavior within 6 months of imprisonment	*r* = −.01
20)	Werner, Rose, Murdach, Yesavage [40]	5 social workers	40 Admission data for psychiatric inpatients	19	Assess imminent violence in the first 7 days following admission	Actual outcome: violent acts in the first 7 days following admission	*r* = .18 (*, +)
21)	Werner, Rose, Yesavage [41]	30: 15 psychologists, 15 psychiatrists	Case material for 40 male patients	19	Predicting patients' violence during the first 7 days following admission	Actual violence during the first 7 days following admission	*r_s_* = .12
	*Psychological science, students:*						
22)	Gorman, Clover, Doherty [42]	8 students	75: *I, III:* 50 interviews, *II, IV:* 25 paper-people	*II, IV:* 6, *I, III:* 12	Prediction of each student's scores in an attitude scale (*I, II*) and a psychology examination (*III, IV*)	Actual data: *I, II:* Attitude scale, *III, IV:* Examination scale (▴)	*I: r_0_* = .23, *II: r_0_* = .05, *III: r_0_* = .46, *IV: r_0_* = .45 (*)
23)	Reynolds, Gifford [43]	*I:* 7 students, *II:* 10 students, *III:* 28 students	Videotapes	I: 7, II: 8, III: 9	To assess the intelligence: I: Audio condition, II: Visual condition, III: Audio plus visual condition	Wonderlic Personnel Test (a brief intelligence test)	*I: r* = .22, *II: r* = .38, *III: r* = .30
24)	Bernieri, Gillis, Davis, Grahe [44]	*I:* 45 students, *II:* 54 students	50 videotaped debates	*I:* 17, *II:* 24	Rapport judgments	Interactants: self-reports context: *I:* Adversarial, or *II:* Cooperative (▴)	*I: r* = .19, *II: r* = .28
25)	Lehman [45]	14 students	Case material for 40 male patients	19	Assess imminent violence in the first 7 days following admission	Actual outcome (violent acts in the first 7 days following admission)	*r* = .24 (*, +)

*Note.* ▴ = subjective criterion. *r_0_* = type of correlation is unknown. (*) = idiographic approach (cumulating across individuals). (*, +) = both research approaches are considered.

^1^ This publication contains two studies.

**Table 2:** Characteristics of studies in the ‘miscellaneous’ domain ordered by expertise.

Study		Number of judges	Number of judgments	Number of cues	Judgment task	Criterion	Research area	Results
*e)*	*Miscellaneous research area, experts:*							
26)	Stewart [46]	7 meteorologists	75 radar volume scans (25)	6	Assess probability of hail or severe hail	Observed event	Meteorology	*r_0_* = .43 (*)
	*Both, experts and students:*							
27)	Stewart, Roebber, Bosart [47]	4: 2 students, 2 experts	*I:* 169 forecast days	12	24-h maximum temperature forecasts 12-h minimum temperature forecasts	*I, II:* Actual temperature	Meteorology	*I: r_0_* = .96
			*II:* 178 forecast days	13				*II: r_0_* = .96
			*III:* 149 forecast days	24	12-h precipitation forecasts	*III, IV:* Actual precipitation		*III: r_0_* = .74
			*IV:* 150 forecast days	24	24-h precipitation forecasts			*IV: r_0_* = .71 (*, +)
	*Students:*							
28)	Steinmann, Doherty [48]	22 students	192: 2 sessions with 96 judgments	2	To decide from which of two randomly chosen bags a sequence of chips had been drawn	A hypothetical “judge” (▴)	Other	*r_o_* = .65 (*)
29)	MacGregor, Slovic [49]	*I:* 25 students, *II:* 25 students, *III:* 26 students, *VI:* 27 students	*I – IV:* 40 runner profiles	4	Estimation of the time to complete a marathon	Actual time to complete the marathon	Sport	*I: r* = .42, *II: r* = .63, *III: r* = .39, *VI: r* = .49
30)	McClellan, Bernstein, Garbin [50]	26 psychology students	128 experimental stimuli	5	Magnitude estimations of fins-in and fins-out Mueller Lyer stimuli	Actual magnitude of fins-in and fins-out Mueller Lyer stimuli	Perception	*r_0_* = .72
31)	Trailer, Morgan [51]	75 students	50 situations in a questionnaire	11	Predicting the motion of objects	Actual motion	Intuitive physics	*r_0_* = .15 (*, +)

*Note.* ▴ = subjective criterion. *r_0_* = type of correlation is unknown. (*) = idiographic approach (cumulating across individuals). (*, +) = both research approaches are considered.

### The Psychometric Meta-Analytical Approach

Several studies contributed to the eventual development of various meta-analytical approaches in the 1970s (e.g., [15], [52], [53]). For example, Eysenck [52] concluded from a narrative review that psychotherapy was ineffective, prompting a response from the experienced therapist Glass, who statistically compared the outcomes of psychotherapy and refuted Eysenck's conclusion ([54], see also [55]). Since then, researchers have used meta-analysis to systematically summarize the outcomes of multiple studies to increase the generalizability of results (e.g., regarding the effectiveness of psychological, pedagogical and behavioral interventions [56]; regarding predictors of student achievement [57]).

The meta-analytical approach has undergone continuous development, resulting in a number of approaches such as the Hedges-Olkin [58], the Rosenthal-Rubin [59] and the Hunter-Schmidt [16] approach (for an overview, see [60], [61]; for a critical discussion, [62]). Field [63], [64] evaluated different traditional meta-analytical approaches and favored the random-effect model of the Hunter-Schmidt approach. The random-effect model takes into account that the studies included in a meta-analysis are drawn from a greater ‘population’ of studies. Hence, differences in effect sizes across studies arise from sources *within* as well as *between* studies. The traditional, ‘bare bones’ Hunter-Schmidt approach (as evaluated by Field) corrects for sampling error: Since meta-analysis is generally based on many studies with different sample sizes, sampling error is inherent in the data (larger for smaller sample sizes). The Hunter-Schmidt approach has since been additionally modified to correct for up to 11 other methodological artifacts (‘psychometric Hunter-Schmidt approach’; [16], p. 35). Since multiple methodological artifacts threaten the estimations of the *LME* parameters, we argue that the psychometric Hunter-Schmidt is the most appropriate approach for the current study, since it is the only meta-analytical approach that corrects for *multiple* differences in study design.

With regards to potential bias due to measurement artifacts, the knowledge component (*G*) is attenuated by the unreliability of the estimate of the judge, the unreliability of the criterion and the restriction of range in both. Therefore, the bias inherent in estimates of the knowledge component (*G*) can be corrected when *S* (restriction or enhancement of range), the reliability of the judge (see r_tt_^Rs^) and the reliability of the criterion (see r_tt_^Re^) are known. The knowledge component can thus be described as in Equation 3:
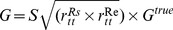
(3)Neglecting the nonlinear knowledge term (*C*) in Equation 1 and considering it as an error term *e*, substituting Equation 3 into Equation 1 results in Equation 4:
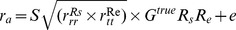
(4)Therefore the unbiased estimate of the knowledge component (*G*) corrected for attenuation and restriction of range would be Equation 5:
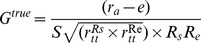
(5)In Equation 5, the psychometric Hunter-Schmidt approach incorporates the estimation of the population parameter according to Wittmann [65], [66]. This equation serves as an illustration of how to psychometrically meta-analyze the *LME* in our study. The psychometrically-corrected component (e.g., *G*) is called “true” and is an approximation of the value without any study design artifact. The “true” value is for example the actual judgmental achievement or the knowledge component without any artifacts introduced by the study design. Put simply, Equation 5 can be divided into three parts.

Firstly, the numerator of the fraction, the term *e*, represents sampling error. Meta-analysis carried out for the purpose of population estimation is often based on different studies including different numbers of participants, which results in sampling errors. Such a sampling error is larger for smaller sample sizes and can be positive or negative. It should be noted that traditional bare-bones meta-analysis corrects *only* for sampling error, although several additional study design artifacts (as introduced) are known. Due to the bias related to sampling error, there is a risk to over- or to underestimate the particular component.

Second, the first part in the denominator describes psychometric concepts of the reliability associated with judges and tasks. Failure to correct for the *reliability of tasks or judges* introduces two dangers that may result in an underestimation of the component. In addition, failure to correct for selection problems, known either as *restriction* or as *enhancement of range* might lead to under- or overestimation of for example judgmental achievement as maybe an extremely easy or difficult task.

Third, in the second part in the denominator, the term *R_s_R_e_*, can be traced back to Brunswik's research and the *LME* (see Equation 2) and represents construct reliability. Wittmann [67], [66], further extended Hunter-Schmidt's psychometric approach by adding the *symmetry* concept. Judgmental achievement increases if both the judgment and the criterion are measured at the same level of aggregation (i.e., they are ‘symmetrical’). For example, if a physician is asked to judge whether cancer is present and the criterion is whether a cancer tumor was detected, then the judgment is not symmetrical, as cancer can exist without a detectable tumor. In contrast, if a physician is asked to judge whether there is cancer only when a cancer tumor has been detectable, then the judgment and the criterion are said to be symmetrical. We did not control for symmetry in the current analysis. Neglecting symmetry may lead to two additional risks of potentially underestimating the components.

To summarize, due to the potential for different methodological artifacts, there is a tendency to over- or underestimate the “true value” of each component as illustrated by Equation 5. Based on Equation 5, the odds of underestimating the component with a bare-bones meta-analysis are 6 (sampling error, reliability of tasks or judges, selection effects, symmetry of tasks, judges) to 2 (sampling error, selection effects) as compared with estimates generated from a psychometric meta-analysis.

In our psychometric Hunter-Schmidt meta-analysis, we weighted each judgment task by the number of judges to correct for sampling error. To correct for measurement error with regards to both the criterion and human judgment, we used an artifact distribution compatible with the Hunter-Schmidt approach ([16], p. 137). To correct for measurement error on the judgment side within medicine and business, we use the studies' reliability values (e.g., [36]) or, otherwise, the retest reliabilities provided by Ashton [68] who reported retest reliability values across and within different domains. For example, when a study within the medical domain did not report measurement reliability, we used the mean of the reported test-retest reliability of .73 to correct for measurement error. No area specific retest-reliability values were available for measurement error correction by judges in the areas of education, psychology or miscellaneous professions. We therefore used the Reliability Generalization approach [69] to correct the measurement error of judges in these areas. In line with the Reliability Generalization theory, we estimate a retest-reliability value for our measurement error corrections, namely .90, as an upper bound of the reliability distributions, as the averaged retest-reliability of professional judgments across domains is .78 (see [68]). Hence, our assumed measurement-error may have led to an underestimation of all components as we assume a smaller measurement error relative to the average reported by Ashton [68]. With regards to the measurement reliability values on the ecological side of the lens model (i.e., the criterion for against which human judgment is compared), we distinguished between three types of criteria. First, for subjective judgments, e.g., a physician's judgment (see [25]); we used the same approach as with the judgment side of the model as previously described. Second, for test criteria (e.g., MMPI), we used the test-specific retest-reliability value as available in the literature. Third, we did not correct objective criteria (e.g., an angiography; see [24]), as we assumed that there is only minimal measurement error with objective criteria. Finally, we considered further artifacts, such as the dichotomization of a continuous variable (see [38]).

**Figure 3:**
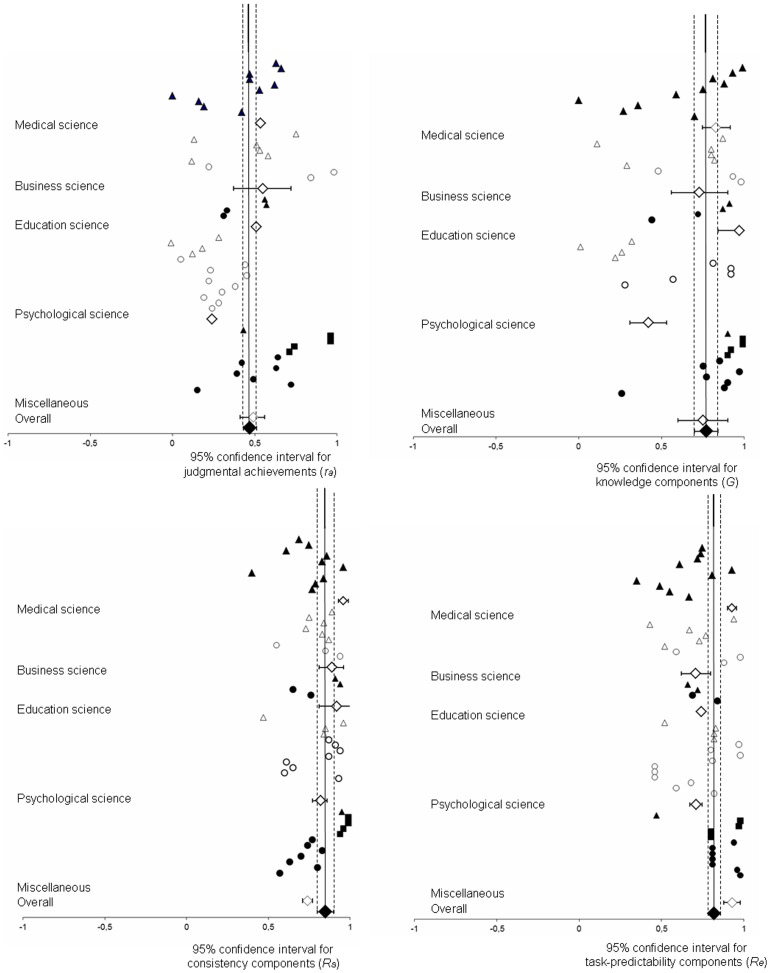
Forest plots of judgmental achievement and the underlying components.

Forest plots (see Figure 3) provide an overview of the results of the included studies and psychometrically corrected confidence intervals (see [16], p. 207). We also report credibility intervals as an indication of the existence of moderators of judgmental achievement. In contrast to confidence intervals, credibility intervals are calculated with standard deviations *after* removing artifacts. If the credibility interval includes zero or is sufficiently large, then there is a higher potential for moderator variables relative to when the credibility interval is small and excludes zero. Hunter and Schmidt [16] also recommend a simple 75% rule to detect moderator variables, which is typically more accurate than significance tests used to assess homogeneity. According to this rule, if the variance after correcting for artifacts accounts for less than 75% of the uncorrected variance (i.e., when artifacts account for less than 25% of the total variance, moderator variables are suspected). It should be noted that the variance remaining after artifact correction represents the upper boundary of any potential moderator effects, as it is impossible to correct of all potential artifacts. We emphasize that we do not apply Fisher-Z transformations, in line with the recommendations of Hunter and Schmidt [16].

Finally, we apply the trim-and-fill method introduced by Duval and Tweedie [70] to estimate a possible publication bias in order to check the robustness of our estimations. By applying the trim-and-fill method, we estimated the effect sizes of potentially missing studies and included them in a further psychometric meta-analysis corrected for publication bias. In the following, we refer to this approach that to our knowledge is hereby introduced to the literature for the first time as the *psychometric trim-and-fill method*. We use the retest-reliability values to correct for judgment reliability, as in the case of education and psychology, and we assume no measurement error on the criterion side.

## Results

Tables 3 to 6 and Figure 3 display the results of the meta-analyses. The results of the bare-bones meta-analysis for each research area are displayed first, followed by the results of the psychometric meta-analysis. Whenever the psychometrical trim-and-fill method did not match the psychometric results with regards to the indication of moderators, the suggested values are reported as publication bias in the tables.

**Table 3:** Comparison of estimations of judgmental achievement (r_a_) with different meta-analytical approaches ordered by domain and experience level.

			Bare-bones meta-analysis					Psychometric meta-analysis				
						*Credibility interval*					*Credibility interval*	
Domains, experience levels	*k*	*N*	*r_a_*	*var_corr_*	75%	.10	.80	*r_a_*	*var_corr_*	75%	.10	.80
Medical science^a^	10	258	.40	.00	157.00	.40	.40	.53	.00	170.93	.53	.53
Business science	9	239	.50	.07	24.45	.16	.83	.55	.09	24.45	.16	.93
*Publication bias*	*13*	*332*						.*22*	.*31*	*13.56*	*−.50*	.*93*
Experts	6	116	.36	.00	87.73	.36	.36	.40	.00	87.73	.40	.40
*Publication bias*	*9*	*136*						.*27*	.*05*	*60.24*	*−.01*	.*55*
Students	3	123	.63	.10	8.52	.22	1.00	.70	.11	8.52	.27	1.12
Education science	4	156	.39	.00	177.89	.39	.39	.51	00	355.11	.51	.51
*Publication bias*	*5*	*176*						.*41*	.*02*	*74.99*	.*22*	.*59*
Experts	2	40	.57	.00	975.69	.57	.57	.62	.00	975.69	.62	.62
Students	2	116	.33	.00	27,143	.33	.33	.55	.00	82,558	.55	.55
b	2	116						.36	.00	27,136	.36	.36
Psychology	14	249	.22	.00	448.50	.22	.22	.24	.00	448.54	.24	.24
Experts	4	59	.10	.00	975.77	.10	.10	.11	.00	975.77	.11	.11
Students	10	190	.26	.00	606	.26	.26	.29	.00	607.07	.29	.29
Miscellaneous	12	249	.44	.02	67.55	.25	.62	.49	.02	67.55	.31	.67
Experts	5	15	.65	.00	401.61	.65	.65	.68	.00	401.61	.68	.68
*Publication bias*	*7*	*23*						.*31*	.*00*	*158.46*	.*31*	.*31*
Students	11	234	.43	.00	86.40	.43	.43	.48	.00	86.55	.48	.48
*Publication bias*	*16*	*276*						.*35*	.*07*	*53.59*	.*01*	.*68*
Overall	49	1,151	.39	.02	69.42	.21	.57	.45	.02	74.55	.27	.63
Experts	27	488	.37	.00	129.00	.37	.37	.47	.00	135.00	.47	.47
Students	26	663	.40	.02	58.94	.21	.58	.46	.02	64.20	.27	.64

*Note. k* = Number of correlations (tasks) according to Hunter and Schmidt [16]. *N* = Total sample size according to Hunter and Schmidt [16]. *r_a_* = mean true score correlation according to Hunter and Schmidt [16]. *var_corr_* = corrected variation according to Hunter and Schmidt (2004, variance of true score correlation). 75% rule = Percentage variance of observed correlations due to all artifacts, if below 75%, it indicates moderator variable. – mean true score correlation increased the value of 1.

^a^ In medical science only experts are included.

^b^ we reran the analysis and substituted the .09 value with a .90 value.

**Table 4:** Comparison of estimations of the linear knowledge component (G) with different meta-analytical approaches ordered by domain and experience level.

			Bare-bones meta-analysis					Psychometric meta-analysis				
						*Credibility interval*					*Credibility interval*	
Domains, experience levels	*k*	*N*	*G*	*var_corr_*	75%	.10	.80	*G*	*var_corr_*	75%	.10	.80
Medical science^a^	10	258	.61	.02	50.72	.42	.79	.82	.02	68.47	.64	1.00
Business science	9	239	.66	.07	15.85	.32	.99	.73	.08	15.85	.37	1.09
Experts	6	116	.55	.05	33.56	.26	.83	.60	.06	35.56	.28	.91
Students	3	123	.78	.05	6.95	.49	1.0	.86	.06	6.95	.54	1.17
Education science	4	156	.73	.01	35.62	.60	.85	.81	.01	35.62	.66	.85
Experts	2	40	.89	.00	313.80	.89	.89	.98	.00	313.80	.98	.98
Students	2	116	.68	.00	51.03	.68	.68					
Psychology	9	105	.38	.02	73.77	.19	.56	.42	.03	73.77	.19	.64
*Publication bias*	*11*	*121*						.*27*	.*13*	*45.46*	*−.19*	.*73*
Experts	4	59	.17	.00	444.93	.17	.17	.18	.00	444.93	.18	.18
Students	5	46	.65	.03	57.65	.42	.87	.72	.04	57.65	.46	.97
*Publication bias*	*7*	*62*						.*41*	.*21*	*35.14*	*−.17*	.*99*
Miscellaneous	12	249	.68	.07	19.26	.34	1.00	.75	.08	19.27	.38	1.11
*Publication bias*	*17*	*313*						.*54*	.*20*	*17.90*	*−.03*	*1.11*
Experts	5	15	.92	.00	768.55	.92	.92	.96	.00	768.55	.96	.96
Students	11	234	.66	.06	24.15	.34	.97	.74	.08	24.15	.37	1.1
Overall	44	1,007	.63	.05	24.91	.34	.91	.77	.07	37.11	.43	1.1
Experts	27	488	.57	.04	43.69	.31	.82	.71	.06	49.80	.39	1.0
Students	21	519	.69	.04	21.81	.43	.95	.77	.06	22.00	.45	1.0

*Note. k* = Number of correlations (tasks) according to Hunter and Schmidt [16]. *N* = Total sample size according to Hunter and Schmidt [16]. *G* = mean true score correlation according to Hunter and Schmidt [16]. *var_corr_* = corrected variation according to Hunter and Schmidt ([16]., variance of true score correlation). 75% rule = Percentage variance of observed correlations due to all artifacts, if below 75%, it indicates moderator variable. – mean true score correlation increased the value of 1.

^a^ In the medical science only experts are included.

**Table 5:** Comparison of estimations of the consistency component (R_s_) with different meta-analytical approaches ordered by domain and experience level.

			Bare-bones meta-analysis					Psychometric meta-analysis				
						*Credibility interval*					*Credibility interval*	
Domains, experience level	*k*	*N*	*R_s_*	*var_corr_*	75%	.80	.10	*R_s_*	*var_corr_*	75%	.80	.10
Medical science^a^	10	258	.81	.00	74.95	.81	.81	.96	.00	126.87	.96	.96
Business science	9	239	.81	.01	28.60	.68	.93	.89	.02	28.60	.70	1.0
Experts	6	116	.62	.00	268.23	.62	.62	.69	.00	108.29	.69	.69
Students	3	123	.77	.03	12.68	.54	.99	.85	.03	12.68	.62	1.00
Education science	4	156	.73	.00	43.52	.60	.85	.93	.00	554.87	.93	.93
*Publication bias*	*6*	*196*						.*67*	.*20*	*22.80*	.*09*	*1.25*
Experts	2	40	.92	.00	1,241	.92	.92	.96	.00	1,241	.96	.96
Students	2	116	.66	.00	422.27	.66	.66	1.00	b			
Psychology	12	150	.79	.01	71.34	.66	.91	.83	.01	71.34	.07	.96
Experts	4	59	.85	.01	48.83	.72	.98	.89	.01	48.83	.76	1.0
Students	8	91	.74	.00	107.28	.74	.74	.78	.00	107.28	.78	.78
*Publication bias*	*11*	*115*						.*59*	.*09*	*35.15*	.*21*	.*97*
Miscellaneous	12	249	.71	.00	90.86	.71	.71	.75	.00	90.92	.75	.75
*Publication bias*	*17*	*272*						.*67*	.*05*	*34.01*	*−.23*	1.5
Experts	5	15	.95	.00	1,724	.95	.95	.98	.00	1,724	.98	.98
*Publication bias*	*6*	*19*						.*78*	.*06*	*66.77*	.*46*	1.09
Students	11	234	.69	.00	148.50	.69	.69	.73	.00	148.50	.73	.73
Overall	47	1,052	.77	.00	53.34	.64	.90	.85	.00	100.63	.85	.85
*Publication bias*	58	1,260						.66	.14	17.31	.18	1.13
Experts	27	488	.83	.00	89.61	.83	.83	.92	.00	138.60	.92	.92
*Publication bias*	*29*	*496*						.*90*	.*01*	*52.26*	.*77*	1.02
Students	24	564	.71	.01	75.37	.63	.80	.78	.00	139.48	.78	.78
*Publication bias*	*33*	*664*						.*61*	.*11*	*24.43*	.*18*	*1.18*

*Note. k* = Number of correlations (tasks) according to Hunter and Schmidt [16]. *N* = Total sample size according to Hunter and Schmidt [16]. *R_s_* = mean true score correlation according to Hunter and Schmidt [16]. *var_corr_* = corrected variation according to Hunter and Schmidt ([16]., variance of true score correlation). 75% rule = Percentage variance of observed correlations due to all artifacts, if below 75%, it indicates moderator variable.

^a^ In medical science only experts are included.

^b^ mean true score correlation increased the value of 1.

**Table 6:** Comparison of estimations of the task-predictability component (R_e_) with different meta-analytical approaches ordered by domain and experience level.

			*Bare-bones meta-analysis*					*Psychometric meta-analysis*				
						*Credibility Interval*					*Credibility Interval*	
Domains, experience level	*k*	*N*	*R_e_*	*var_corr_*	75%	.10	.80	*R_e_*	*var_corr_*	75%	.10	.80
Medical science^a^	10	258	.67	.00	105.89	.67	.67	.92	.00	198.55	.92	.92
Business science	9	239	.71	.02	34.97	.52	.89	b				
Experts	6	116	.62	.00	108.29	.62	.62	b				
Students	3	123	.79	.02	13.91	.60	.97	b				
Education science	4	156	.70	.00	257.26	.70	.70	.74	.00	257.26	.74	.74
Experts	2	40	.68	.00	1,690	.68	.68	.72	.00	1,690	.72	.72
Students	2	116	.71	.00	145.93	.71	.71	.75	.00	145.93	.75	.75
Psychology	14	249	.68	.00	77.79	.68	.68	.72	.00	78.62	.72	.72
*Publication bias*	*16*	*265*						.*64*	.*05*	*32.73*	.*35*	.*92*
Experts^b^	4	59	.80	.00	256.36	.80	.80	b				
Students	10	176	.63	.00	91.12	.63	.63	.68	.00	91.12	.68	.68
*Publication bias*	*13*	*220*						.*54*	.*09*	*27.90*	.*15*	.*94*
Miscellaneous	12	249	.88	.01	23.75	.75	1.00	.93	.01	23.74	.80	1.00
Experts^b^	5	15	.69	.00	356.44	.69	.69	b				
Students	11	234	.89	.00	39.67	.89	.89	.94	.00	39.67	.94	.94
Overall	49	1,151	.73	.01	44.21	.60	.85	.81	.01	66.00	.69	.93
Experts	27	488	.68	.00	126.13	.68	.68	.76	.00	157.52	.76	.76
*Publication bias*	*32*	*23*						.*59*	.*06*	*36.54*	.*27*	.*90*
Students	26	663	.77	.02	31.23	.18	.58	.81	.02	31.45	.62	.99

*Note. k* = Number of correlations (tasks) according to Hunter and Schmidt [16]. *N* = Total sample size according to Hunter and Schmidt [16]. *R_e_* = mean true score correlation according to Hunter and Schmidt [16]. *var_corr_* = corrected variation according to Hunter and Schmidt ([16], variance of true score correlation). 75% rule = Percentage variance of observed correlations due to all artifacts, if below 75%, it indicates moderator variable. – mean true score correlation increased the value of 1.

^a^ In medical science only experts are included.

^b^ see bare-bones meta-analysis, no correction because this category includes only objective criterions.

### Judgmental Achievement

Table 3 and Figure 3 show the meta-analytic results of judgmental achievement. Correcting for sampling error (bare bones approach) only results in an estimated judgmental achievement of .39. Correcting for additional artifacts with the psychometric approach resulted in an increased estimate of .45. That is, across all included lens model studies, human judgment correlated .45 with the given criterion.

### Domain and Expertise as Moderators

The relatively small reduction in variability resulting from the psychometric approach relative to the bare bones approach suggested the existence of moderator variables under the assumption of no measurement error on the criterion side for objective criteria. We therefore re-ran the analyses within each domain (medicine, business, education, psychology, miscellaneous), for experts versus novices, and for expertise within domain (e.g., expert teachers versus novice teachers). These subsequent analyses revealed that judgmental achievement depended on decision domain. Specifically, judgmental achievement was lowest in psychology (*r_a_* = .22) and higher in education (*r_a_* = .39), medicine (*r_a_* = .40), miscellaneous professional domains (*r_a_* = .44), and highest in business (*r_a_* = .50). The results from the psychometric meta-analysis confirmed this pattern of results.

Against our expectation, results indicated that students reached a slightly higher judgmental achievement than experts. The 75% rule and the credibility intervals indicated the existence of moderator variables among student's judgmental achievement. We therefore reran our analysis, separating *expertise within domains*. This analysis revealed that the potential for moderator variables (once again as indicated by the 75% rule as well as by the credibility intervals) amongst experts runs not across all domains. In contrast, the analysis indicated the existence of moderator variables amongst business science students only.

Inspection of the scatter plots of students' judgmental achievement within the business domain indicated that Wright's study [32] had low values of judgmental achievement and might have influenced our results. Excluding this study from the sample increased estimated judgmental achievement (*r_a_* = .97, *var_corr_* = .00), but still indicated the presence of moderator variables according to the 75% rule (30.51%).

Finally, the application of the psychometric trim-and-fill method generally confirmed our results. However, estimates of judgmental achievement among business experts dropped to a low value (no publication bias was indicated in studies using business students). Likewise, experts' judgments in other research domains decreased from .68 to .31. The application of the psychometric fill-and-trim method to judgmental achievement in the field of education indicated the existence of moderator variables. The potential for moderator variables according to the credibility intervals and the 75% rule decreased after we separated the analysis by experience level in the education domain. We therefore assume that experience level is a moderator variable within education. The judgment-achievement values for students in other domains remained stable after correcting for potential publication bias.

### Components of Judgmental Achievement

Tables 4 to 6 and Figure 3 present the estimates of the *LME* parameters. As seen in Table 4, our results indicated high values of the knowledge component (*G*) in nearly every domain/experience-level except among experts in psychology. In addition, the results from the psychometric trim-and-fill method suggested a lower value for students' knowledge components. Hence, it seems that our analysis overestimated the knowledge component *(G)* among students, although the knowledge component for students was lower relative to experts.

Table 5 displays estimates of the consistency component (*R_s_*). The results from the bare-bones and psychometric meta-analyses both suggest high values and generally indicate no moderator variables for all analyses across domains and expertise-level. All of the estimated consistency components (*R_s_*) remain high when using the psychometric fill-and-trim method. In addition, the results from the psychometric fill-and-trim method indicated the existence of moderators within education science, among experts in the miscellaneous domain, and aggregated cross all domains.

Finally, Table 6 presents estimates for the task predictability component (*R_e_*). All values were above .68 in each and every analysis across domains and experience-level. The 75% rule indicated moderator variables across all domains, mainly based on students' task predictabilities in business science and the miscellaneous domain. In addition, the psychometric trim-and-fill method suggested that task predictabilities were overestimated amongst psychology students, as the 75% rule suggested the existence of moderators.

### The Success of Bootstrapping Judges with a Linear Model

Table 7 compares the success of bootstrapping judges with a linear judgment model (see Equation 2) based on corrected versus uncorrected estimates of *LME* parameters. Failure to correct the component estimates for various artifacts clearly lead to underestimations of bootstrapping success. Indeed, the current results with corrected parameters indicate that the linear judgment models are actually more successful than previous studies have suggested (see [2], [6]). Hence, using corrected estimations of the *LME* components (e.g., *G*, *R_e_*) has practical consequences for the success of bootstrapping with linear judgment models. We therefore argue that corrected parameter estimates should be used to evaluate the success of bootstrapping.

**Table 7:** Comparison of the success of bootstrapping judges with a linear judgment model (GR_e_) based on different meta-analytical approaches (bare-bones vs. psychometric approach).

Research area	*k*	*N*	Bare-bones (*GR_e_*)	Psychometric (*GR_e_*)	Estimated success (Bare-bones)	Estimated success (Psychometric)
Medical	10	258	.41	.76	.01	.36
Business	9	239	.47	.52^b^	−.03	.02
Educational	4	156	.51	.72	.12	.22
Psychological^a^	9	105	.26	.30	.04	.08
Miscellaneous	12	249	.60	.70	.16	.26
Overall^a^	44	1007	.46	.62	.07	.23

^a^ = there are 5 more *R_e_* values of studies included having 144 participants;

b = component *R_e_* is not corrected.

## Discussion

The major finding of our study is that bare-bones meta-analysis (e.g., [5], [6], see one-trial category), clearly underestimates true judgmental achievement values relative to psychometric meta-analysis, which more appropriately corrects for study design artifacts. Consequently, we argue that a psychometric meta-analysis is needed to more accurately evaluate judgment accuracy and can help researchers to more efficiently detect moderators. So far, previous meta-analyses of lens model studies have neglected the need to correct for multiple artifacts, although even minor increases in judgmental achievement may have a high practical impact at the individual level, for example, in life or death decisions in the medical domain. Our results indicate that failure to correct for artifacts (as with a bare-bones meta-analysis) leads to underestimations of all *LME* parameters across and within expertise domains, and the potential for moderator variables is generally overestimated. Parameter estimates from psychometric meta-analysis can be used to improve linear judgment models and hence bootstrapping, especially in areas where the price of false decision-making is high.

With regards to specific moderators of judgmental achievement, the present study confirms the pattern previously found for comparisons *between* different domains [5], namely, that judgmental achievement varies greatly across the medical, educational, psychological, business and other professional domains. In line with the meta-analysis of Aegisdottir et al. (p. 368) [1], we found low judgmental achievement in psychological science, for example, in the prediction of violence. Our analysis revealed that such low judgmental achievement within psychology may be explained by a moderate knowledge component. Hence, the question arises whether judgmental achievement in psychology can be improved by increasing the knowledge component, meaning that psychologists would need to expand their relevant knowledge for linear information integration. The success of psychometrically-corrected linear judgment models was higher than the low human judgmental achievement in psychology. Therefore, it might be particularly worthwhile to bootstrap judges within this domain (for further information, see [71]).

Against our expectation, the results of the meta-analyses suggest that experts do not make much better judgments than non-experts at the aggregated level. However, the effect of expertise appears to depend on domain. Specifically, within the business and psychology domains, students had higher judgmental achievement than experts. This surprising result may imply situations of learning and feedback (see also [22]). That is, higher judgmental achievement among experts relative to students may indicate higher feedback and learning in the respective domain. It seems possible to improve judgmental achievement through feedback and learning. There is only one study [47], however, that directly compares experts and students in four different tasks. Our results and conclusions regarding this point should therefore be taken with caution.

An innovative aspect of the current study was that we estimated publication bias using a psychometric trim-and-fill method, potentially leading to better estimates. To the best of our knowledge, calculation of publication bias has previously only been applied within bare-bones meta-analyses (see [72]), and we are not aware of any previous psychometric meta-analysis that has corrected for publication bias in this way. We recommend that researchers check the robustness of the results of future psychometric meta-analyses by using the psychometric trim-and-fill method described in this paper. We caution, however, that the psychometric trim-and-fill method used in the current study may need improvement and replication, because the underlying data were heterogeneous, which can potentially be problematic. Indeed, Rothstein [73] asserted that disentangling the effects of publication bias from other sources of heterogeneity can be difficult.

As common in meta-analytical research, the studies included in the analyses did not always report all of the data needed to calculate “true” judgmental achievement values (e.g., measurement reliability). Indeed, researchers interested in conducting psychometric meta-analyses often face the problem of missing data. Based on the Reliability Generalization theory [69], we suggest estimating a measurement error with an *rr* = .9 to check the robustness of the data as a possible solution. We also emphatically recommend that future researchers thoroughly and consequently report all relevant information on study method and results (e.g., reliability values, dichotomizations) in order to enhance the accuracy of further meta-analyses (and hence their usefulness). We would also like to encourage researchers to report more idiographic data in lens model studies (see [21]). For instance, multi-level analysis (see [74]) could be applied to gain further knowledge about judges' strategies within and between tasks.

In the current study, we corrected for a number of methodological artifacts (sampling error, measurement error, and dichotomization). Importantly, there may well be additional artifacts for which we did not correct. On this note, we heartily agree with Hunter and Schmidt [16] that, “all quantitative estimates are approximations. Even if these estimates are quite accurate, it is always desirable to make them more accurate, if possible” (p. 168). For instance, Wittmann [66], [67], further extended Hunter-Schmidt's psychometric approach by adding the symmetry concept. We did not control for symmetry in the current analysis. Hence, we may have underestimated overall judgmental achievement, although our analyses rarely indicated any moderator variables, suggesting that there is not much variance left for further artifact correction.

In the current study, we focused on the evaluation of the success of bootstrapping with only linear judgment models. However, we did not consider experience within domains in detail. Further analyses are needed to shed light on this topic (see [75]).

As linear judgment models are often criticized for lack of user friendliness, we also see our analysis as an inspiration for the development of new judgment models (see [76]). The true power of psychometrically corrected linear judgment models should urgently be evaluated against new kinds of judgment models.

In sum, our study demonstrates that psychometric meta-analysis is useful for evaluating judgmental achievement and for constructing better linear judgment models for bootstrapping. This first psychometric meta-analysis of lens model studies confirms and extends previous results from bare-bones meta-analysis: Judgmental achievement clearly varies across domains. Our analysis also extended previous research on the potential moderating role of expertise within and between decision domains. The current analysis revealed that failure to correct for methodological artifacts can lead to underestimations of judgmental achievement and overestimations of heterogeneity between studies. Consequently, the success of bootstrapping with linear judgment models is also underestimated if *LME* parameters are not corrected for methodological artifacts. We therefore recommend that future researchers follow a psychometric approach in order to arrive at less biased estimations and more successful linear judgment models. If the relevant data for psychometric analyses (e.g., data on measurement error) are not immediately available, researchers can conduct robustness analysis with estimated values.

## Supporting Information

Checklist S1 **PRISMA Checklist for systematic review and meta-analysis.**
(DOC)
